# Behavior Change Techniques Used in Digital Behavior Change Interventions to Reduce Excessive Alcohol Consumption: A Meta-regression

**DOI:** 10.1093/abm/kax029

**Published:** 2018-01-27

**Authors:** Claire V Garnett, David Crane, Jamie Brown, Eileen F S Kaner, Fiona R Beyer, Colin R Muirhead, Matthew Hickman, Emma Beard, James Redmore, Frank de Vocht, Susan Michie

**Affiliations:** 1 Research Department of Behavioural Science and Health, UCL, UK; 2 Research Department of Clinical, Educational and Health Psychology, UCL, UK; 3 Institute of Health and Society, Newcastle University, UK; 4 School of Social and Community Medicine, University of Bristol, UK

**Keywords:** Behavior change techniques, Alcohol, Drinking, Digital interventions, Meta-regression, Systematic review

## Abstract

**Background:**

Digital behavior change interventions (DBCIs) appear to reduce alcohol consumption, but greater understanding is needed of their mechanisms of action.

**Purpose:**

To describe the behavior change techniques (BCTs) used in DBCIs and examine whether individual BCTs, the inclusion of more BCTs or more Control Theory congruent BCTs is associated with increased effectiveness.

**Methods:**

Forty-one randomized control trials were extracted from a Cochrane review of alcohol reduction DBCIs and coded for up to 93 BCTs using an established and reliable method. Random effects unadjusted and adjusted meta-regression models were performed to assess associations between BCTs and intervention effectiveness.

**Results:**

Interventions used a mean of 9.1 BCTs (range 1–22), 23 different BCTs were used in four or more trials. Trials that used “Behavior substitution” (−95.112 grams per week [gpw], 95% CI: −162.90, −27.34), “Problem solving” (−45.92 gpw, 95% CI: −90.97, −0.87) and “Credible source” (−32.09 gpw, 95% CI: −60.64, −3.55) were significantly associated with greater alcohol reduction than trials without these BCTs. The “Behavior substitution” result should be treated as preliminary because it was reported in only four trials, three of which were conducted by the same research group. “Feedback” was used in 98% of trials (*n* = 41); other Control Theory congruent BCTs were used less frequently: for example, “Goal setting” 43% (*n* = 18) and “Self-monitoring” 29%, (*n* = 12).

**Conclusions:**

“Behavior substitution,” “Problem solving,” and “Credible source” were associated with greater alcohol reduction. Many BCTs were used infrequently in DBCIs, including BCTs with evidence of effectiveness in other domains, such as “Self-monitoring” and “Goal setting.”

## Teaser

Digital behaviour change interventions for alcohol that used “Behaviour substitution”, “Problem solving” and “Credible source” were associated with greater alcohol reduction than those interventions without them.

## Introduction

Brief interventions to reduce harmful and hazardous alcohol consumption in the UK are often delivered in primary care settings to nontreatment seeking populations [1]. They are cheaper than more extensive interventions and, for patients not needing treatment, can be more acceptable and as effective [2–4]. Nonetheless, clinicians have been reluctant to deliver brief interventions despite their demonstrated effectiveness, citing lack of resources, insufficient training, excess workload, lack of financial incentives and fear of antagonizing patients as barriers [5, 6]. Barriers to implementing brief interventions may also arise from patients who are concerned about the stigma associated with excessive alcohol consumption, have negative experiences with or expectancies about treatment, believe the problem is not severe enough to warrant treatment, or are worried about their privacy [7, 8]. Gaining access to treatment can be difficult for some patients [9], especially those in rural areas [10].

Digital behavior change interventions (DBCIs), or e-Health interventions [11, 12], are those delivered on websites, by email or through mobile phones. DBCIs can address the cost, time and training barriers experienced when delivering brief interventions in person [13, 14]. Their convenience and anonymity may further reduce patient barriers to seeking help [15, 16]. Systematic reviews and meta-analyses have found DBCIs can result in small but meaningful reductions in alcohol consumption, which could have important public health implications given their low cost and broad reach [17–26]. However, the heterogeneity of intervention content has led to calls for greater understanding of the mechanisms of action that contribute to intervention effectiveness [21, 24, 27, 28].

### Identifying the Mechanisms of Action of Interventions

Reliably specifying the behavior change techniques (BCTs) used in interventions allows their active ingredients to be identified, evidence to be synthesized, interventions to be replicated and more effective interventions to be developed [29, 30]. A taxonomy of 93 distinct BCTs (BCTTv1), grouped into 16 categories, has been developed by consensus methods with input from a large group of international behavior change experts [30]. A BCT is “an observable, replicable, and irreducible component of an intervention designed to alter or redirect causal processes that regulate behavior; that is, a technique is an ‘active ingredient’ (e.g. ‘Feedback,’ ‘Self-monitoring,’ and ‘Reinforcement’)” (pp. 82, [30]). Taxonomies have been used to identify the BCTs in physical activity and dietary smartphone applications (apps) [31, 32], alcohol reduction apps [33], wearable activity monitors [34] and internet-based health interventions [26], and have helped systematic reviews progress from treating complex interventions as a homogenous group [35]. Applying the BCTTv1 taxonomy to DBCIs evaluated in RCTs, and assessing associations of included BCTs with effectiveness, could allow the mechanisms of action of DBCIs to be identified, which would be beneficial for future intervention developers.

A recent assessment of the BCTs in alcohol reduction DBCIs by Black et al. [36] reported that better outcomes were associated with use of the BCTs of “Commitment,” “Social comparison,” “Feedback,” and “Review of goals,” and worse outcomes were associated with the BCT of “Providing information on the consequences of alcohol consumption.” However, Black et al. included trials with respondents reporting only moderate consumption, as well as trials where participants were mandated to take part. It is important to determine whether findings generalize when only trials of hazardous or harmful drinkers are included and/or trials where individuals mandated to participate are excluded; as the effectiveness of BCTs may be moderated by levels of alcohol consumption, and the motivation to reduce consumption may be greater in people who are not mandated to participate in the intervention. Determining which BCTs are most effective for hazardous and harmful drinkers is particularly important as they are responsible for the majority of health, economic and social costs resulting from alcohol consumption [37]. The current meta-regression specified interventions using the 93-item taxonomy BCTTv1, rather than the 42-item alcohol-specific one used by Black et al. The 93-item taxonomy was developed across behavioral domains, building on domain-specific taxonomies, including the alcohol one. It is more comprehensive, includes more developed definitions and allows BCTs to be compared across different areas of behavior change. The wider taxonomy is also more fine-grained, for example, “Feedback” is elaborated into separate BCTs of “Feedback on behavior,” “Feedback on outcome(s) of behavior” and “Biofeedback” (e.g. blood alcohol content level), and consequently allows for more specific understanding of the mechanisms of action of interventions.

Behavior change interventions are often complex and consist of a number of BCTs [38], which may act additively, synergistically, or counteract each other [39, 40]. Just as atoms interact to form different molecules, the effectiveness of interventions may be a product of how different BCTs combine. To understand which combinations of BCTs are likely to be effective, we need to turn to theory. Theories provide an analytical framework through which understanding can be gained not just of whether an intervention was effective but also how and why it was effective [41, 42]. Control Theory is one such framework that provides a model of self-regulation for behavior change to occur [43] and is, therefore, a promising theory for health behavior change interventions. Control Theory states that behavior is goal-driven and feedback about a discrepancy between current behavior and a goal leads to behavioral adjustments [43]. The BCTs congruent with Control Theory are: “Goal-setting,” “Self-monitoring,” “Feedback,” “Review of goals,” and “Action planning.” Brief interventions to reduce excessive alcohol consumption that included self-monitoring were associated with larger effect sizes [44]. Systematic reviews of other domains have found that interventions including more than one of the BCTs congruent with Control Theory resulted in increased physical activity and healthy eating [39, 40] and promoted goal attainment [45] more than interventions that only included one technique. Therefore, in addition to examining the BCTs within DBCIs, we will investigate whether interventions including more Control Theory congruent BCTs are associated with greater effectiveness in reducing alcohol consumption.

This article reports a BCT analysis of all the interventions included in the primary analysis of a Cochrane review of the effectiveness of digital interventions for reducing hazardous and harmful alcohol consumption in community-dwelling populations (systematic review registration number: CRD42015022135). The aims of this article are to: (a) describe the BCT content of DBCIs to reduce alcohol consumption; (b) identify whether individual BCTs are associated with the effectiveness of alcohol reduction DBCIs; (c) examine whether the inclusion of a larger number of BCTs is associated with increased intervention effectiveness; and (d) examine whether interventions that include more Control Theory congruent BCTs are associated with increased effectiveness.

## Methods

### Design

Trials included in a Cochrane review of DBCIs for alcohol reduction [46] were analyzed using meta-regression. Cochrane reviews are considered a gold standard for high-quality information. Meta-regression is a particularly helpful approach for understanding causes of heterogeneity across divergent interventions and study methodologies. The additional contribution of this meta-regression was to reliably code the BCTs of included interventions. Associations between the inclusion of BCTs and effect size across trials were assessed with unadjusted and adjusted meta-regression models.

### Identification and Selection of Trials

Trials selected for inclusion in the Cochrane review were identified by searching electronic databases in health, social science, psychology, education, and human-computer interaction for RCTs of DBCIs to reduce excessive alcohol consumption. Additional searches were performed on relevant web sites considered likely to contain evaluations of DBCIs, for example, the International Alcohol Information Database, Beacon 2.0 and Drug and Alcohol Findings. Databases and sites were searched for terms such as: alcohol drinking; alcohol use; risks; internet; computers; smartphone. Full details of the search strategy and the review protocol are published elsewhere (systematic review registration number: CRD42015022135) [46].

### Inclusion and Exclusion Criteria

Trials were included if they were RCTs primarily delivered through a computer or mobile device, directly targeted hazardous and/or harmful drinkers and aimed to reduce alcohol consumption or harm. A control condition must have been included though comparisons with face-to-face interventions were excluded from this meta-regression. Trials were excluded if they were directed mainly towards people seeking specialist treatment for their alcohol consumption, or if the intervention was delivered in a secondary or tertiary care setting as this Cochrane review was focused on prevention and a narrower population.

### Measures

The outcome variable was the mean difference in the quantity of alcohol consumed in a specified time period between intervention and control for each included trial. For trials that did not report it directly, the outcome data on quantity of alcohol consumed was converted to grams per week (conversion factors reported elsewhere [46]). For trials with more than one control or experimental arm and where these arms were very similar, results for arms were combined in the meta-analysis. Where trials reported these data at more than one follow-up time-point, we used data from the longest follow-up. The exposure variable was whether an intervention included a BCT (dummy coded as 1 = present or 0 = absent for each BCT), the number of BCTs in an intervention, or the number of Control Theory congruent BCTs in an intervention.

### Procedure

Studies were reviewed initially on their title and abstract, and then the full research paper of any study identified as potentially eligible. This review procedure was conducted independently by two researchers and discrepancies were resolved by discussion or consulting a third researcher, if necessary. Duplicate papers reporting data from the same trial were identified by two researchers (F.B. and C.M.) and the secondary papers were excluded before data extraction. Data extraction included details on the intervention and was conducted for all included studies by two researchers; a standardized data extraction form that had been piloted was used.

All trials were coded for BCTs using an established and reliable method [30]. Authors of all included trials were contacted for supplementary materials that may further explain intervention content, which was also coded for BCTs. Intervention descriptions were read line-by-line, text that may indicate the presence of a BCT was highlighted, and highlighted text was compared with the definition for the BCT given in the taxonomy [30]. A BCT had to be explicitly present to be coded as included.

The reliability of the method was assessed and improved in iterative rounds of coding. In the first step, two coders (D.C. and C.G.) independently coded a sample of five trials. Coding differences were resolved through discussion and the coding manual was reviewed and updated in the light of these discussions. If agreement could not be reached, the views of a behavior change expert were sought. Inter-rater reliability was assessed with both the Kappa (κ) and PABAK statistics. Cohen’s Kappa accounts for coders agreeing on the presence of codes [47]. PABAK is an adjusted Kappa statistic that accounts for coders agreeing on the presence and the absence of codes [48]. While it is important to measure levels of agreement about the absence of BCTs, using PABAK alone could exaggerate levels of agreement when coding against a taxonomy of 93 BCTs, the large majority of which were likely to be absent in any one intervention [31, 40]. Therefore, inter-rater reliability was assessed with both statistics. The first round of joint coding led to an inter-rater reliability of κ = 0.73, PABAK = 0.95, which reflects a substantial level of agreement according to Landis and Koch’s [47] definition. As this exceeded our pre-determined threshold of κ = 0.70, remaining trials were coded by one coder, with the second coding 33% (13/40) of the same trials to ensure against rater drift. The inter-rater reliability for all included trials that were joint coded was κ = 0.73, PABAK = 0.96. The data extracted was not confirmed with the original authors of the trials.

### Analysis

The metareg command in Stata was used to conduct a series of random effects unadjusted meta-regression models to assess the associations between the type, number and combination of BCTs and effect size [49]. Given differences in the recruited samples and designs of the included trials, the assumptions of a fixed effects meta-regression model was assumed to be unlikely to hold. For this reason, a random effects meta-regression model was used to explore variations in alcohol consumption as a function of BCTs. A random-effects meta-regression has the advantage of allowing for residual, unexplained variance in true effects across different trials, that is, for between study variations in effect size. This approach has been recommended previously [50]. The *I*^2^ statistic describes the proportion of total variation in study estimates that is due to heterogeneity [51] and was used to assess the magnitude of heterogeneity. The regression coefficients represent the mean of unstandardized effects between trials that differentially included a BCT in the intervention and those which did not (dummy coded as 1 = present or 0 = absent for each BCT). Each unstandardized effect was the mean difference between intervention and control (expressed in grams of alcohol per week). Only BCTs uniquely present in experimental arms, that is, not present in both experimental and control arms, were included in analysis (BCTs were rarely included in control arms, *M* = 0.73, SD = 1.66). To be included in analysis, each BCT needed to be used in 10% of trials, equivalent to at least four separate trials (a criterion used in a previous meta-regression study of the BCTs contained within physical activity and healthy eating interventions [39]). A negative coefficient for a BCT indicated that trials using that BCT produced a larger pooled effect than trials that did not.

To assess the independent association after mutual adjustment, we created an adjusted meta-regression model including all BCTs with a meaningful association with effect size in the unadjusted models. A meaningful association was defined as *B* > 23, which was the lower confidence interval of the effect size reported in a meta-analysis of the effect of brief advice on alcohol consumption [1]. The associations in the adjusted model were regarded as providing the primary indication of association between BCTs and effect size.

To assess the association between the total number of BCTs included in experimental arms and effect size we created a random effects unadjusted meta-regression model. Lastly, we assessed the overall fit of a model, in terms of adjusted *R*^2^, containing only a theoretically derived cluster of Control Theory congruent BCTs. These BCTs were grouped into four categories: Goals: “Goal setting (behavior),” “Goal setting (outcome),” “Review behavior goal(s),” “Review outcome goal(s),” “Discrepancy between current behavior and goal”; Self-monitoring: “Self-monitoring of behavior,” “Self-monitoring of outcome(s) of behavior,” “Monitoring of emotional consequences”; Feedback: “Feedback on behavior,” “Feedback on outcome(s) of behavior,” “Biofeedback” and Action plans: “Action planning.” Trials were dummy coded as 1 = used BCTs from three or four of these groupings; or 0 = used BCTs from two or fewer of these groupings.

## Results

### Study Selection and Characteristics

**Fig. 1.**
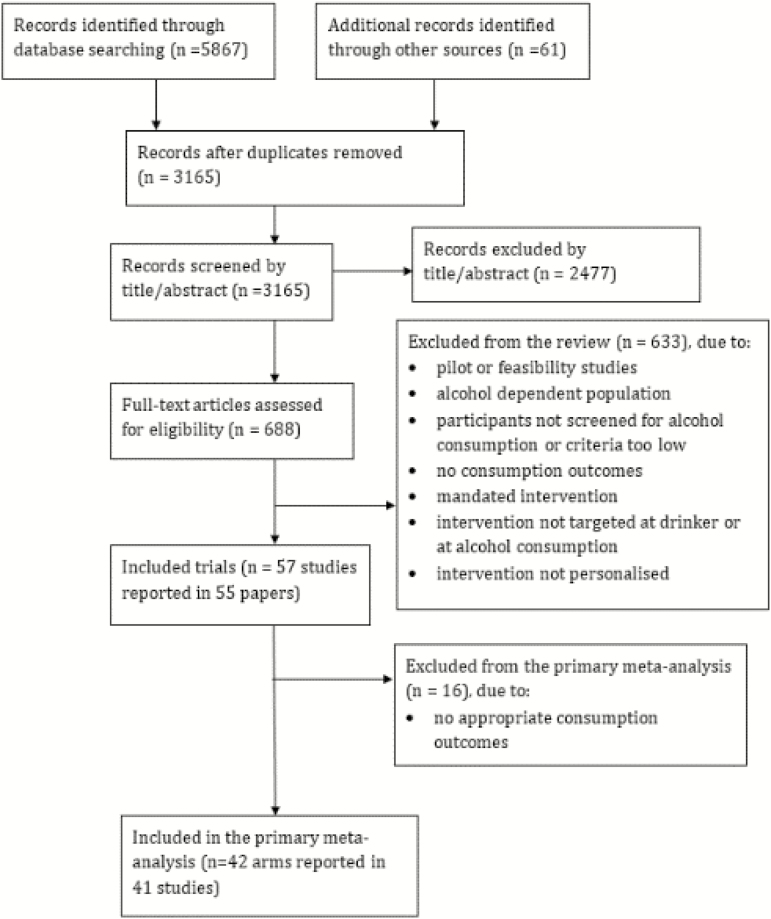
Flow chart of trials. Reproduced with permission from: Personalized digital interventions for reducing hazardous and harmful alcohol consumption in community-dwelling populations [52].

The search strategy was performed up to September 2015 and identified 3,165 records after removing duplicates. Forty-one trials met the inclusion criteria, reported appropriate information for inclusion in the primary meta-analysis and were coded for BCTs (Fig. 1). Of the included trials, 11 authors (27%) could not be contacted, 7 (18%) reported that there was no supplementary materials to send and 22 authors (55%) sent supplementary materials, which were also coded for BCTs.

Of the 41 trials, 23 took place in North America, 15 took place in Europe and three in Australasia. Seventeen trials recruited people of any age and 24 recruited students or people aged under 25. The majority of interventions were web-based (*n* = 34, 83%), six (15%) comprised a stand-alone computer programme, and one (2%) was a smartphone app. Control conditions included no intervention (screening only or screening/assessment); written information about alcohol effects (electronic or printed) and/or consumption recommendations or other health-related advice (not alcohol – as an attention control). The longest follow-up period for each trial ranged from one (*n* = 8) to 12 (*n* = 7) months. One of the 41 trials contained two experimental arms which resulted in 42 comparisons between experimental and control arms. See Supplementary Table 1 for the characteristics of the included studies.

### BCT Content of DBCIs to Reduce Alcohol Consumption

The five most frequently used BCTs uniquely present in experimental arms were: “Feedback on behavior” (85.7%, *n* = 36), “Social comparison” (81.0%, *n* = 34), “Information about social and environmental consequences” (71.4%, *n* = 30), “Feedback on outcomes of behavior” (69.0%, *n* = 29) and “Social support (unspecified)” (64.3%, *n* = 27) (Table 1).

**Table 1:** Frequency of Behavior Change Techniques (BCTs) Present in Digital Behavior Change Interventions (DBCIs)

BCTs	% (*n*)
*2. Feedback and monitoring*	
2.2. Feedback on behavior	85.7% (36)
2.7. Feedback on outcome(s) of behavior	69.0% (29)
2.6. Biofeedback	50.0% (21)
2.3. Self-monitoring of behavior	26.2% (11)
2.4. Self-monitoring of outcome(s) of behavior	14.3% (6)
*5. Natural consequences*	
5.3. Information about social and environmental consequences	71.4% (30)
5.2. Salience of consequences	50.0% (21)
5.1. Information about health consequences	33.3% (14)
5.6. Information about emotional consequences	7.1% (3)
5.4. Monitoring of emotional consequences	4.8% (2)
*1. Goals and planning*	
1.2. Problem solving	33.3% (14)
1.4. Action planning	31.0% (13)
1.1. Goal setting (behavior)	28.6% (12)
1.3. Goal setting (outcome)	11.9% (5)
1.6. Discrepancy between current behavior and goal	11.9% (5)
1.5. Review behavior goal(s)	4.8% (2)
1.7. Review outcome goal(s)	2.4% (1)
1.8. Behavioral contract	2.4% (1)
*3. Social support*	
3.1. Social support (unspecified)	64.3% (27)
3.2. Social support (practical)	16.7% (7)
3.3. Social support (emotional)	2.4% (1)
*6. Comparison of behavior*	
6.2. Social comparison	81.0% (34)
*4. Shaping knowledge*	
4.1. Instruction on how to perform the behavior	52.4% (22)
4.2. Information about Antecedents	14.3% (6)
4.4. Behavioral experiments	2.4% (1)
*9. Comparison of outcomes*	
9.2. Pros and cons	35.7% (15)
9.1. Credible source	31.0% (13)
*8. Repetition and substitution*	
8.2. Behavior substitution	9.5% (4)
8.1. Behavioral practice/rehearsal	2.4% (1)
8.4. Habit reversal	2.4% (1)
8.7. Graded tasks	2.4% (1)
*12. Antecedents*	
12.2. Restructuring the social environment	9.5% (4)
12.3. Avoidance/reducing exposure to cues for the behavior	7.1% (3)
*10. Reward and threat*	
10.3. Nonspecific reward	4.8% (2)
10.9. Self-reward	4.8% (2)
10.4. Social reward	2.4% (1)
10.6. Nonspecific incentive	2.4% (1)
*15. Self-belief*	
15.4. Self-talk	9.5% (4)
15.1. Verbal persuasion about capability	2.4% (1)
15.3. Focus on past success	2.4% (1)
*7. Associations*	
7.1. Prompts/cues	7.1% (3)
*11. Regulation*	
11.2. Reduce negative emotions	7.1% (3)
*13. Identity*	
13.2. Framing/reframing	2.4% (1)
*14. Scheduled consequences*	
14.2. Punishment	2.4% (1)
*16. Covert learning*	

The BCTs present in the trials are grouped taxonomically and listed in order of the most frequently coded BCT for each taxonomic group.

Of the 93 possible BCTs that could have been used, 15 were used in more than 20% of trials, 44 were used at least once and 49 were never used. The mean number of BCTs used in experimental arms was 9.2 (SD=5.3), the median was 9 and the range 1–22.

The prevalence of Control Theory congruent BCTs was as follows: Goals: “Goal setting (behavior)” (28.6% of trials, *n* = 12), “Goal setting (outcome)” (11.9%, *n* = 5), “Discrepancy between current behavior and goal” (11.9%, *n* = 5), “Review behavior goal(s)” (4.8%, *n* = 2), “Review outcome goal(s)” (2.4%, *n* = 1). Self-monitoring: “Self-monitoring of behavior” (26.2%, *n* = 11), “Self-monitoring of outcome(s) of behavior” (14.3%, *n* = 6), “Monitoring of emotional consequences” (4.8%, *n* = 2). Feedback: “Feedback on behavior” (85.7%, *n* = 36), “Feedback on outcome(s) of behavior” (69.0%, *n* = 29), “Biofeedback” (50.0%, *n* = 21). “Action planning” (31.0%, *n* = 13).

### Association Between BCTs and Intervention Effectiveness

The primary meta-analysis in the Cochrane review found that participants randomized to a digital intervention group drank a mean of 23.6 (95% CI: 16.0, 31.2) grams of alcohol per week less than controls at end of follow-up [52]. There was considerable heterogeneity in the estimate of the effect size between trials (*I*^2^: 78%).

In unadjusted models (Table 2), the BCTs of “Goal setting” (−43.94 grams per week, *p* = .01, 95% CI: −78.59, −9.30, ${\text{R}}_{\text{adj}}^{2}$: 6.64%), “Problem solving” (−48.03 grams per week, *p* < .01, 95% CI: −77.79, −18.27, ${\text{R}}_{\text{adj}}^{2}$: 25.01%), “Information about antecedents” (−74.20 grams per week, *p* < .01, 95% CI: −117.72, −30.68, ${\text{R}}_{\text{adj}}^{2}$: 32.15%), “Behavior substitution” (−123.71 grams per week, *p* < .001, 95% CI: −184.63, −62.80, ${\text{R}}_{\text{adj}}^{2}$: 48.53%) and “Credible source” (−39.89 grams per week, *p* = .02, 95% CI: −72.66, −7.11, ${\text{R}}_{\text{adj}}^{2}$: 15.60%) were all associated with greater reduced alcohol consumption. No other BCTs were significantly associated with reduced consumption in the unadjusted models. The total number of BCTs present in the intervention was not significantly associated with reduced consumption in the unadjusted models (−2.71 grams per week, *p* = .07, 95% CI: −5.65, 0.23, ${\text{R}}_{\text{adj}}^{2}$: 3.26%).

**Table 2:** Unadjusted Associations Between Behavior Change Techniques (BCTs) and the Unstandardized Effect Size of the Intervention

BCT		MGPW (SE)	*N*	*p*	95% CI	*I* ^2^	Adj *R*^2^
1.1	Goal setting (behavior)	−43.94 (17.14)	12	.01*	−78.59, −9.30	78.05%	6.64%
1.2	Problem solving	−48.03 (14.72)	14	<.01*	−77.79, −18.27	74.64%	25.01%
1.3	Goal setting (outcome)	−14.43 (23.46)	5	.54	−61.85, 32.99	77.71%	−2.95%
1.4	Action planning	−26.21 (16.58)	13	.12	−59.73, 7.30	77.57%	5.45%
1.6	Discrepancy between current behavior and goal	−33.88 (24.97)	5	.18	−84.35, 16.58	78.24%	0.15%
2.2	Feedback on behavior	12.97 (21.30)	36	.55	−30.08, 56.02	78.31%	−7.13%
2.3	Self-monitoring of behavior	−30.39 (17.14)	11	.08	−65.03, 4.26	78.36%	2.07%
2.4	Self-monitoring of outcome(s) of behavior	−8.60 (22.37)	6	.70	−53.81, 36.61	78.52%	−4.67%
2.6	Biofeedback	10.81 (15.24)	21	.48	−19.99, 41.62	77.85%	1.55%
2.7	Feedback on outcome(s) of behavior	−4.62 (16.45)	29	.78	−37.87, 28.63	78.48%	−5.63%
3.1	Social support (unspecified)	−19.55 (15.39)	27	.21	−50.65, 11.55	78.53%	−0.41%
3.2	Social support (practical)	−26.35 (22.59)	7	.25	−72.01, 19.31	77.18%	0.29%
4.1	Instruction on how, perform the behavior	4.46 (15.51)	22	.78	−26.89, 35.80	78.55%	−5.77%
4.2	Information about antecedents	−74.20 (21.53)	6	<.01*	−117.72, −30.68	74.91%	32.15%
5.1	Information about health consequences	16.75 (15.70)	14	.29	−14.99, 48.49	78.42%	0.06%
5.2	Salience of consequences	21.99 (14.86)	21	.15	−8.05, 52.02	78.17%	4.92%
5.3	Information about social and environmental consequences	28.88 (16.56)	30	.09	−4.59, 62.34	77.59%	1.01%
6.2	Social comparison	24.25 (18.95)	34	.21	−14.06, 62.56	78.53%	−4.98%
8.2	Behavior substitution	−123.71 (30.14)	4	<.001*	−184.63, −62.80	72.92%	48.53%
9.1	Credible source	−39.89 (16.22)	13	.02*	−72.66, −7.11	75.84%	15.60%
9.2	Pros and cons	−30.10 (15.77)	15	.06	−61.97, 1.78	77.57%	10.15%
12.2	Restructuring the social environment	−22.91 (31.52)	4	.47	−86.62, 40.79	78.56%	−7.66%
15.4	Self-talk	−41.53 (26.37)	4	.12	−94.84, 11.77	77.93%	6.04%

MGPW (SE) = mean grams per week (standard error). BCTs only included in analysis if present in at least four trials. Results from the standardized model were broadly similar to the unstandardized model; as a result, data are only reported from the unstandardized model.

*p < .05.

In an adjusted model that included BCTs with a *B* > 23 in the unadjusted model, three BCTs—“Behavior substitution” (−95.12 grams per week, *p* = .01, 95% CI: −162.90, −27.34), “Problem solving” (−45.92 grams per week, *p* = .05, 95% CI: −90.97, −0.87) and “Credible source” (−32.09 grams per week, *p* = .03, 95% CI: −60.64, −3.55)—were associated with a reduction in alcohol consumption (Table 3). “Behavior substitution” is defined as “Prompt substitution of the unwanted behavior with a wanted or neutral behavior”; “Problem solving” as “Analyze, or prompt the person to analyze, factors influencing the behavior and generate or select strategies that include overcoming barriers and/or increasing facilitators”; and “Credible source” as “Present verbal or visual communication from a credible source in favor of or against the behavior” [30]. The adjusted meta-regression model produced relatively good indices of fit and reduced heterogeneity (*I*^2^: 67.24%, ${\text{R}}_{\text{adj}}^{2}$: 59.51%, *p* < .01) compared with the heterogeneity of *I*^2^ = 78.0% from the primary meta-analysis [52].

**Table 3:** Adjusted Associations Between Behavior Change Techniques (BCTs) and the Unstandardized Effect Size of the Intervention

BCT		*B* (SE)	*p*	95% CI
1.1	Goal setting (behavior)	0.75 (19.60)	.97	−39.40, 40.89
1.2	Problem solving	−45.92 (21.99)	.05*	−90.97, −0.87
1.4	Action planning	30.75 (19.50)	.13	−9.19, 70.68
1.6	Discrepancy between current behavior and goal	−29.86 (23.97)	.22	−78.97, 19.25
2.3	Self-monitoring of behavior	−6.34 (18.35)	.73	−43.91, 31.24
3.2	Social support (practical)	33.73 (21.85)	.13	−11.03, 78.49
4.2	Information about antecedents	−43.38 (23.93)	.08	−92.39, 5.63
5.3	Information about social and environmental consequences	24.64 (12.17)	.05	−0.30, 49.57
6.2	Social comparison	3.92 (13.11)	.77	−22.93, 30.77
8.2	Behavior substitution	−95.12 (33.09)	.01*	−162.90, −27.34
9.1	Credible source	−32.09 (13.94)	.03*	−60.64, −3.55
9.2	Pros and cons	6.68 (13.68)	.63	−21.33, 34.69
15.4	Self-talk	−8.41 (26.69)	.76	−63.09, 46.27

Only BCTs with *B* > 23 in the unadjusted analysis included in the adjusted analysis.

*p < .05.

A total of 16 trials used BCTs from three or four of the groupings of Control Theory BCTs. These trials were weakly associated with effectiveness (−30.76 grams per week, *p* = .06, 95% CI: −62.35, 0.83) and the model produced a poor index of fit and did not improve the heterogeneity from the primary meta-analysis (*I*^2^ = 78.03%, *R*^2^ = 1.81%).

## Discussion

The BCTs of “Behavior substitution,” “Problem solving,” and “Credible source” were significantly associated with a reduction in grams of alcohol consumed per week in both unadjusted and adjusted random effects models. The mean number of BCTs used in interventions was 9.2 (SD = 5.3), the median was 9 and the range 1–22, and a total of 23 different BCTs were used in at least four trials. By comparison, the mean number of BCTs used in popular alcohol reduction apps was 3.6 [33]. No significant association was detected between the number of BCTs used or use of more Control Theory congruent BCTs and intervention effectiveness.

“Behavior substitution” was associated with a mean reduction in drinking of 12 UK units of alcohol per week. The BCT was present in four trials (one paper reported two trials [53–55]), three of which were performed by the same research group who implemented the technique in the same way; asking participants to: a) Reflect on the pros and cons of drinking; b) Detail the outcomes expected from drinking; and c) Select behaviors that could provide an acceptable alternative to drinking. There is existing evidence suggesting that “Behavior substitution” may be an effective BCT for alcohol reduction [44, 56] and for other health behavior change interventions [57–60]. “Problem solving” was associated with a mean reduction in drinking of six UK units of alcohol per week. The BCT was present in 14 trials [53–55, 61–70], half of which adopted a Relapse Prevention or Coping Planning approach. Relapse Prevention and Coping Planning both aim to promote behavior maintenance by helping people develop proactive strategies for dealing with situations in which relapse might occur. Relapse Prevention additionally aims to help people react positively to situations when relapse has occurred [71, 72]. A notable characteristic of “Problem solving” and “Behavior substitution” is that these BCTs help people who are engaged in self-directed behavior change identify practical and specific ways of reaching their drinking reduction goals. The potential effectiveness of these BCTs suggests that DBCIs may be enhanced when users are given guidance and direction about how to maintain behavior change, particularly when that comes from a source perceived to be credible. “Credible source” was associated with a mean reduction in drinking of four UK units of alcohol per week. The BCT was present in 13 trials [53–55, 64, 67, 73–77] and generally consisted of advice about national guidelines for consumption, or advice about drinking provided by a member of the study. However, a review of governmental policies to reduce alcohol-related harm found that no evaluation of the effectiveness of providing alcohol consumption guidelines had been published [78]. The credibility of a source has consistently been found to affect the persuasiveness of information delivered via traditional media [79, 80]. When health information is delivered online users can be undiscriminating in their assessment of which sources are credible [81, 82]. Further investigation of the effectiveness of providing such information in DBCIs is warranted.

Findings from this study differ to a recent assessment of the BCTs in alcohol DBCIs (Black et al. [36]), which found that the BCTs of “Commitment,” “Social comparison,” “Feedback,” and “Review of goals” were associated with better outcomes, and the BCT of “Providing information on the consequences of alcohol consumption” was associated with worse outcomes [36]. There are at least two reasons why the two trials may have found different BCTs to be associated with effectiveness. Firstly, we used inclusion criteria that particularly related to our populations of interest. Most notably we only included trials where participants were not mandated to take part and where they were known (via a specific screening process) to be drinking at harmful or hazardous levels; whereas Black et al. included mandated participants and all drinkers regardless their level of consumption. As a consequence, only 27 trials analyzed here were also analyzed by Black et al. Secondly, the 93-item taxonomy includes a BCT of “Credible source” (the 42-item taxonomy does not), and has three “Feedback” BCTs, two “Review goal” BCTs and three BCTs that provide information on negative consequences of performing a behavior; whereas the 42-item taxonomy only has one BCT for each. Replication work using the same taxonomy on the same or different data sets may help determine which BCTs are effective in DBCIs and whether there are differences in BCTs that are effective across different populations.

Nearly all trials gave feedback in one form or another. “Feedback on behavior,” which usually consisted of information about levels and/or patterns of drinking, was the most popular BCT. “Feedback on outcomes of behavior, which usually consisted of information about the health risks or other negative consequences that might occur should the participant’s drinking continue at its current level, was the fourth most popular BCT and “Biofeedback,” which consisted of information about recent levels of blood alcohol content, was the eighth most popular BCT. Other frequently used BCTs were: “Social comparison,” which involved presenting a participant with information comparing their drinking with that of their peers; “Information about social and environmental consequences,” which was often coded alongside “Feedback on outcomes of behavior” as interventions commonly provided feedback about the potential negative consequences of the participant continuing to drink at their current level; and “Social support (unspecified),” which often involved the provision of links to services that might further help a participant reduce their drinking.

Some BCTs with evidence of effectiveness in other behavioral domains were used infrequently. For example, “Self-monitoring of behavior,” defined as “Establish a method for the person to monitor and record their behavior(s) as part of a behavior change strategy” [30] has been found effective for a variety of health behaviors [39, 83–88]. A reanalysis of a Cochrane review of face-to-face brief alcohol interventions found that interventions which included self-monitoring were associated with larger effect sizes [44]. However, participants in only a quarter of the digital trials in this review were asked to self-monitor their drinking as part of the intervention, despite the ability of DBCIs to facilitate the easy, ongoing and anonymous recording of consumption. Similarly, the BCTs: “Facilitate goal setting” and “Review of behavioral goals” have been found effective in other health behavior interventions [39, 40] and were frequently used BCTs in face-to-face interventions to reduce excessive alcohol consumption [44]. However, less than a third of trials in this study used the BCT of “Goal setting (behavior),” five trials used the BCT of “Goal setting (outcome)” and only three used either “Review behavior goal(s)” or “Review outcome goal(s).”

The evidence that the use of more BCTs improves intervention effectiveness is mixed. Interventions targeted at lower-income groups which aimed to increase physical activity and/or healthy eating, or reduce smoking were found to be marginally more effective when they contained fewer BCTs [89]. Other reviews have found that interventions for a range of health behaviors which included more BCTs tended to have larger effect sizes [26, 90]. These mixed findings may relate to participant socio-demographic characteristics or differences in the pattern of the health behavior between participants. It is difficult to determine without further research; a factorial trial, in which different groups of participants are given different numbers of BCTs, may be necessary to answer this question empirically.

The finding of a weak association between use of more Control Theory congruent BCTs and intervention effect differs from reviews which found that interventions using more Control Theory BCTs (“Goal-setting,” “Self-monitoring,” “Feedback,” “Review of goals,” and “Action planning”) resulted in greater physical activity and healthy eating [40], increased goal attainment [45] and reduced consumption in face-to-face alcohol interventions [44]. The lack of an effect may reflect the importance of increasing motivation rather than improving self-regulation for people wishing to reduce their consumption of alcohol without face-to-face engagement. Once consumed, alcohol has a negative effect on self-regulation and can result in attention becoming focused on meeting immediate needs rather than long-term goals [91]. The PRIME theory of motivation argues that our actions are determined by what we most want or need at any moment in time, and that new behaviors are enacted only when the motivation to change is strong enough to overcome competing wants and needs at that moment [92]. Therefore, it is possible that increasing the motivation to reduce consumption may be a more effective behavior change strategy for digital interventions without face-to-face engagement than increasing self-regulation skills, which will subsequently be diminished by the consumption of alcohol.

### Strengths and Limitations

To our knowledge, this is the first study to use the comprehensive 93-item BCT taxonomy to examine the effectiveness of BCTs in alcohol reduction DBCIs. Given the increasing number of these interventions and the lack of understanding about their mechanisms of action, our analysis is an important step in the accumulation of evidence on the effectiveness of component BCTs, and the contrast of our findings with Black et al. [36] demonstrates the need for replication. The inclusion of these BCTs within standalone interventions, or as individual modules within a larger intervention, warrants further investigation in an experimental context.

The BCT taxonomy is a comprehensive, hierarchical, reliable and generalizable method for systematic specification, evaluation and implementation of behavior change interventions [30]. The BCT taxonomy can be applied to many different types of intervention and is relevant to a wide range of behaviors [30]. Using a taxonomic method for describing the content of behavior change interventions provides a common language for understanding the intervention content and a foundation for developing more effective interventions to improve health [93]. Of the 93 BCTs in the taxonomy that the trials were coded for, 49 BCTs were not used in any. The least frequently used categories of BCTs were: “Association’s,” “Regulation,” “Antecedents,” “Identity,” and “Scheduled Consequences’ BCTs; with no BCTs from ‘Covert Learning’.” The most frequently used categories of BCTs in DBCIs were “Social Support,” “Goals and Planning,” “Feedback and Monitoring,” “Shaping Knowledge,” “Natural Consequences,” “Comparison of Behavior,” “Comparison of Outcomes” and “Self-belief.” These findings indicate that there are many BCTs available that DBCIs for alcohol reduction are not currently using. This may be due to a lack of empirical or theoretical evidence for their inclusion, and not all 93 BCTs would be appropriate for all behaviors and each mode of intervention delivery. However, it could be that the lack of use of many BCTs in the taxonomy is indicative of a limited design process that did not consider all the possible BCTs that could be selected. This BCT taxonomy as a method for specifying the content of interventions has limitations. A principal limitation being that BCT coding depends on reported content, which is a well-known challenge of using reports as primary data sources [94]. The list of BCTs is not comprehensive and requires continuous improvement in the form of future versions.

One major limitation with meta-regression is that study characteristics are often highly correlated leading to issues with multicollinearity [49]. There was some evidence in this study that BCTs tended to cluster together in papers. However, other indicators of possible multicollinearity were not present: there was no evidence of extremely large standard errors or large changes in coefficients caused by the deletion or addition of BCTs [95], correlations between all BCTs were <0.7, and variance inflation factors were <4. The total number of BCTs included in the final multivariate meta-regression was also small which limits collinearity effects. Nonetheless, caution should be taken when interpreting the coefficients directly [95]. In view of these limitations, some researchers may judge the pattern of results in the unadjusted models as most important.

In addition, failure to identify evidence for associations should not be taken as evidence for the null hypothesis of no effect. A nonsignificant effect may be due to the available data being insensitive to detect an effect, rather than evidence for no effect [96]. The small number of interventions available for analysis and the infrequent use of many BCTs (70 of the 93 BCTs were used less than four times) meant that the possible effects of most BCTs could not be evaluated. It is unclear whether BCTs are missing because they were not reported as included or because they were not included in the intervention in the belief that they were not useful. Other BCTs were used so frequently (“Feedback on behavior” and “Social comparison” were both present in more than 80% of trials) as to reduce the ability to assess their association with effectiveness. The potential for robust conclusions is also limited by the modest sample size and by the quality of reporting; recognized issues with the incomplete reporting of intervention content [97] may have resulted in BCTs being incorrectly coded as present or absent. This introduces noise, increases the potential for bias due to misclassification and undermines the power to test associations.

To resolve this, authors should be encouraged to report intervention content in sufficient detail for accurate coding of BCTs to be achieved, using BCTTv1 to allow for comparison across behavioral domains. Moreover, simply recording a BCT as present or absent does not take into account the frequency, intensity or form in which it was delivered. The form of a DBCI, that is, the way the intervention is presented, its ability to meet user needs and the user experience that results from its use [98] are likely to play an important role in the depth and length of user engagement with a BCT. Greater understanding of the “dose” [99] of a BCT, its quality [100] and how it acts in combination with other BCTs is needed in order to fully evaluate its effectiveness. The relationship described by a meta-regression is an observational association across trials, any identified association with one characteristic of the trial may in reality reflect a true association with other correlated characteristics, whether these are known or unknown. The finding of the effectiveness of the BCT of “Behavior substitution” should be treated as preliminary, as the BCT was only used in four trials, three of which reported large effects and were conducted by the same research group using the same implementation. Greater understanding of the effectiveness of this BCT across settings and in other domains and interventions is required to support the generalizability of this finding.

There are likely to be moderators of intervention efficacy, other than BCTs, that might account for variability in effect size. For example, length of follow-up for each trial may have had a moderating effect on intervention effectiveness. The longest follow-up period for each trial was chosen for this study though this meant that it varied between trials. However, in the main Cochrane review, the trials were classified based on their longest follow-up and the change per month of follow-up in the difference in alcohol consumption between experimental and control arms was not statistically significant [52]. Other potential moderators of intervention effectiveness, such as age and gender, have been analyzed and reported in the main Cochrane review [52] and whether reported use of theory in the intervention can account for any of the heterogeneity in intervention effectiveness is analyzed in a separate paper [101]. These moderator analyses were not part of the pre-specified analysis plan and would likely over parametrize the models in the current study, whose main focus was explanatory.

With the limitations of the current literature in terms of the number of trials that met the inclusion criteria for this study, there was not sufficient power to assess potential moderating effects of person-level characteristics on the association between intervention effectiveness. However, this study is a starting point and as more trials are published, it may be possible to conduct further analyses on potential moderating effects. A collaboration between behavioral, computer and system architects—the Human Behavior-Change Project—aims to use machine learning to answer the question of “what works, how well, for whom, in what setting, for what behaviors – and why?” [102]. This will create an up-to-date evidence base to allow researchers to make inferences between behavioral domains, overcoming issues relating to limited trials within a specific behavioral domain.

Another limitation of this study is the generalizability of these findings to different types of digital interventions available. The majority of digital interventions included in this study were web-based with only one of the 41 trials using a smartphone app. The speed of development in technology is much faster than in academic research and therefore it is likely that over the coming years there will be an increase in the number of smartphone apps being used as a DBCI which could be included in any updated review.

## Conclusions

The BCTs of “Behavior substitution,” “Problem solving,” and “Credible source” as reported in intervention descriptions were associated with stronger effectiveness of DBCIs to reduce alcohol consumption and warrant further investigation in an experimental context. Other BCTs, such as “Self-monitoring,” “Goal setting,” and “Review of behavioral/outcome goals,” were rarely used in the trials included in this review, despite good evidence of effectiveness in other behavior change domains. Future DBCIs should be designed using the BCT taxonomy alongside empirical and theoretical evidence for the effectiveness of individual BCTs to facilitate future synthesis and possibly enable more effective interventions to be developed.

## Supplementary Material

Supplementary material is available at *Annals of Behavioral Medicine* online.

Supplementary Materials
