# Supplementary material for: Behavior Change Techniques Used in Digital Behavior Change Interventions to Reduce Excessive Alcohol Consumption: A Meta-regression
Source: Ann Behav Med. 2018 Jan 27;52(6):530–43. doi: 10.1093/abm/kax029 (PMC6361280; doi:10.1093/abm/kax029)
Supplement: Supplementary Materials [file kax029_suppl_supplementary_materials.docx]

Supplementary Table 1: Characteristics of the included studies

| Citation [full reference] | Setting, Population (n; mean age; % male) | Duration of follow-up/months (loss to follow-up) | Intervention | Comparison treatment | Number of BCTs |
| --- | --- | --- | --- | --- | --- |
| Bendtsen 2015 [1] | Sweden, students (n=1605) | 2 (>30% in both groups. Differential loss to follow-up between groups) | AMADEUS-2, accessed via an email link and delivered in a single session. Participants received (i) immediate feedback summarising weekly intake, frequency of heavy episodic drinking and highest blood alcohol concentration; (ii) traffic light graphic of their risk level; (iii) normative feedback comparing their consumption to other Swedish university students. | Waiting list control group. Received no assessment or intervention until 2 months. | 11 |
| Bertholet 2015 [2] | Switzerland, aged 19-21, recruited from army recruitment centres (n=737; 20.75; 100%) | 6 (around 10%) | Web-based intervention comprising seven components: (1) normative feedback; (2) feedback on consequences; (3) calorific value of consumption; (4) blood alcohol consumption for maximum binge episode; (5) indication of risk level; (6) information on alcohol and health; (7) recommendation for low risk drinking. | Control group. No feedback given following initial assessment. | 7 |
| Blankers 2011 [3] | Netherlands, recruited from a substance Abuse Treatment Centre website (n=205; 42.2; 50%) | 3 (>30%) | SAO (Self-help Alcohol Online) web-based intervention that was available across multiple platforms. Participants were encouraged to engage on a daily basis over a period of 4 weeks for 20 minutes per session. The programme comprised '4 Piers': (1) monitored participants' alcohol consumption, helped them set drinking goals and identify risky situations that might lead to relapse; (2) provided feedback on current alcohol consumption and compared this to their drinking goal; (3) focused on building skills and knowledge around coping with craving, drinking lapses, peer pressure, and maintaining motivation in risky situations; (4) provided social support via a web-based forum. | Wait-listed, assessed at 3 months and then received the digital intervention. | 12 |
| Brendryen 2013 [4] | Norway, recruited from online newspaper advertisements (n=244; 67%) | 6 (>20% and differential loss to follow-up between groups) | Balance, a web-based intervention combining both brief and intensive self-help interventions. (1) Screening and feedback session based on personalised normative feedback. Participants identified as risky drinkers were recommended to sign-up for the intensive self-help intervention. (2) The intensive self-help intervention comprised 62 online sessions taking up to 10 hours over 6 months. | Received an e-booklet, issued by the Norwegian Directorate of Health, which provided general information on alcohol and the potential risks and harms of drinking. Neither the screening session nor the booklet contained advice on how to achieve a change in drinking behaviour. | 19 |
| Brief 2013 [5] | USA, participants were army veterans recruited via advertisements on Facebook, aged 18-65 (n=600; 87%) | 2 (>30%; participants with higher consumption were more likely to drop out) | Web-based VetChange intervention involving 8 modules based motivational, cognitive-behavioural, and self-control training strategies; (1-3) Included personalised feedback on their drinking and Post-traumatic stress disorder (PTSD) symptoms, evaluated the importance of and readiness to change, set drinking goals, developed a change plan, and reviewed moderation or abstinence strategies; (4) introduced participants to external high risk situations (i.e. social situations, environmental reminders of combat) and helped them to develop coping plans to manage these situations; (5-7) focused on helping veterans learn a combination of cognitive and behavioural strategies to manage a range of internal high-risk situations for drinking; (6-7) encouraged participants to select topics most relevant to their personal situation; and (8) focused on building a support system to assist with recovery efforts following completion of VetChange. VetChange was delivered over a period of 8 weeks, each session lasts 20 minutes. | Received a delayed intervention. This commenced at the 8-week post-intervention stage of the immediate intervention group; we used only 8 week data when the control group has received nothing. | 18 |
| Butler 2009 [6] | USA, students (n=114; 35%) | 1 (around 20%) | Provided with personalised feedback regarding their use of alcohol but did not have any contact with a clinician. A research assistant seated the participants in a private room and instructed them to review their feedback via computer in the form of a self-paced slide presentation. On average, the session lasted 11.11 mins (SD 3.56) | Completed the pre-intervention assessment battery and met the inclusion criterion but did not receive personalized feedback before completing the follow-up measures. | 8 |
| Chiauzzi 2005 [7] | USA, students (n=265; 19.9; 46%) | 3 (around 20%) | Received the web-based MSB: Alcohol intervention. Rate Myself (based on the BASICS model) was the centerpiece of the site, comprising 4 sets of questions: (1) beliefs regarding alcohol; (2) lifestyle issues; (3) drinking risks; (4) drinking consequences. Participants received immediate tailored feedback based on their responses, with the option of printing out a personal report. In addition, MSB: Alcohol offered: variety of college-specific articles, strategies and interactive tools related to alcohol and drinking on campus; weekly updates of peer stories (Student Voices); Ask the Expert (answers from a college alcohol expert to frequently asked alcohol questions); and college health news. An emergency area helped participants to recognize effective ways to deal with alcohol poisoning and find local resources in the event of urgent medical problems. The intervention was delivered as 1 x 20 minute session over a period of 4 weeks. | Compared the educational content found at various websites. Participants visited websites and read research-based articles about the effects of excessive drinking once a week over 4 consecutive weeks Unlike MSB: Alcohol, the control condition did not involve any tailored, interactive, motivational, or skill-building elements. | 5 |
| Collins 2014 (DBF) [8] | USA, students aged 18-25 (n=366) | 12 (around 20%) | Web-based personalised Decisional Balance Feedback on the advantages and disadvantages of current drinking behaviour based on self-report responses to a baseline decision-balance worksheet comprising: (1) graphs of decision balance proportion; (2) graph and text representations of quantitative total; (3) qualitative content of advantages /disadvantages of current drinking behaviour; (4) likelihood and importance of each advantage/disadvantage. | Received web-based assessment only. After assessment, participants were shown a screen that thanked them for their time and reminded them they would be contacted in 1 month for follow-up. | 1 |
| Collins 2014 (PNF) [8] | USA, students aged 18-25 (n=358) | 12 (around 20%) | PNF (Personalised Norms Feedback) designed to reduce over-estimated perceptions about drinking in their peer group. This comprised 4 main feedback elements: (1) typical weekly quantity of perceived v actual gender peer norms; (2) typical and peak estimated BAL v gender norms; (3) calories consumed from alcohol in a typical week v gender norms; (4) money spent on alcohol during a typical week v gender norms. | Received web-based assessment only. After assessment, participants were shown a screen that thanked them for their time and reminded them they would be contacted in 1 month for follow-up. | 5 |
| Cunningham 2009a [9] | Canada, recruited from an ongoing population telephone survey (n=185; 40.1; 53%) | 6 (around 10%) | Web-based Change Your Drinking (CYD) intervention modelled on Drinker’s Check-up and Fostering Self-Change. Core CYD elements were (1) normative feedback and (2i) summary of the participant’s severity of alcohol problems. In total the CYD intervention tool under 10 minutes to complete. | Did not receive any feedback but were sent a list of the informational components that could be included in a computerised summary for drinkers, asked to consider how useful they might find the different components, and reminded that they would be asked for their opinions at the 3-month follow-up. | 9 |
| Delrahim-Howlett 2011 [10] | USA, recruited via Women Infant and Children (WIC) Special Supplemental Nutrition Clinics, aged 18-45 (n=150; 26.33; 0%) | 1 (around 10%) | Adapted version of the e-CHUG (e-CHeckUp to Go) intervention, tailored to fit the reading and comprehension levels of participants in this trial (high-risk women). Participants were given personalised feedback on alcohol consumption, health risks associated with unhealthy alcohol consumption (general and specific to women of child-bearing age), and social norms. Participants were also provided with tips for sensible drinking and contact information for local support services. | Received printed generic (non=personalised) information post-assessment. The 2-page information sheet covered: alcohol consumption; US Surgeon General's recommendations on alcohol use for women of childbearing age; generic information about foetal alcohol syndrome; and details of local alcohol and other health behaviour resources. | 10 |
| Doumas 2010 [11] | USA, student athletes recruited via the National Collegiate Athletics Association seminar group, aged 18-20 (n=113; 18.08; 43%) | 3 (<10%) | Received the web-based e-CHUG intervention. Participants first completed an online assessment consisting of basic demographic details and information on alcohol consumption, drinking behaviour, and alcohol related consequences. Immediately post-assessment, individualised feedback was provided in the following domains: (1) summary of quantity and frequency of drinking; (2) graphical comparison of their drinking to US adult and college drinking norms; (3) estimated risk status for negative consequences / problematic drinking based on AUDIT scores; (4) genetic risk/ tolerance; (5) approximate financial costs of drinking in the past year; (6) normative feedback comparing individual perception of peer drinking to actual university normative data; (7) and referral information for local agencies In total, the intervention took approximately 30 minutes | Received generic (non-personalised) information only, including facts about alcohol and alcohol consumption, and guidelines on dealing with someone who has had too much alcohol to drink. Participants were asked to "surf the website" for 30 minutes in total. | 6 |
| Doumas 2011 [12] | USA, students recruited from summer orientation sections, aged 17-19 (n=350; 18; 35%) | 3 (>70%) | Web-based e-CHUG intervention. Participants first completed an online assessment consisting of basic demographic details and information on alcohol consumption, drinking behaviour, and alcohol related consequences. Immediately post-assessment, individualised feedback was provided in the following domains: (1) summary of quantity and frequency of drinking; (2) graphical comparison of their drinking to US adult and college drinking norms; (3) estimated risk status for negative consequences / problematic drinking based on AUDIT scores; (4) genetic risk/ tolerance; (5) approximate financial costs of drinking in the past year; (6) normative feedback comparing individual perception of peer drinking to actual university normative data; (7) and referral information for local agencies. In total, the intervention took approximately 30 minutes. | Received an assessment only and were sent an e-mail to access e-CHUG after the intervention phase was completed. | 6 |
| Ekman 2011 [13] | Sweden, students recruited via email (n=654, 42%) | 6 (>70%) | Received e-SBI (electronic Screening and Brief Intervention); they were screened for alcohol use, and received brief feedback consisting of three statements summarising: (1) weekly alcohol consumption; (2) frequency of heavy episodic drinking; and (3) highest BAC in past 3-months; alongside a comparison of the respondents' drinking patterns with safe levels set by the Sweidsh Institute for Public Health. In addition, the intervention group received more extensive normative feedback, with information comparing their alcohol use with peers at the university, and, where applicable, advice on reducing unhealthy levels of consumption. This personalised advice consisted of 12 possible statements of suggestions about the students alcohol habits. | Screened for alcohol use, and received brief feedback only. As above, this consisted of three statements summarising: (1) weekly alcohol consumption; (2) frequency of heavy episodic drinking; and (3) highest BAC in past 3-months; alongside a comparison of the respondents' drinking patterns with safe levels set by the Swedish Institute for Public Health. | 3 |
| Gajecki 2014 [14] | Sweden, students recruited via email (n=1932; 24.7; 48%) | 2 (>20%; differential attrition between arms) | Accessed the web-based Partyplanner app via smartphone for an estimated 7-week period. The app enabled users to (1) plan their drinking in advance to a certain estimated blood alcohol concentration (eBAC), and to later compare their actual alcohol consumption to the app's simulation; (2) as a standalone option, to perform real-time registration to monitor their eBAC levels without prior planning. Colour-coded feedback indicated eBAC levels as drinking events progressed. | Did not receive any intervention and did not have any contact in between baseline and 7-week follow-up assessments. | 6 |
| Geisner 2015 [15] | USA, students aged 18-24 with depressed mood (n=339; 20.14; 38%) | 1 (<10%) | Received alcohol intervention: personalised feedback with a normative component for 5 weeks. Through the feedback, (1) users could compare the frequency and quantity of their own drinking to both perceived and actual drinking norms among college students; (2) protective strategies against problematic alcohol use were suggested; (3) a brief psycho-educational component was also provided, illustrating the potential link between alcohol and depressed mood, but no mood symptoms were targeted by the intervention. This intervention was modelled on social norms approaches and psychoeducation. | Received no interventions or personalised feedback but were directed towards Internet-based information resources on substance abuse and depression. | 5 |
| Hansen 2012 [16] | Denmark, respondents to the Danish Health Examination Survey and were invited by email (n=1380; 58.8; 55%) | 12 (around 20%) | Received computer-based personalised brief advice via the Internet in one session. (1) Participants were informed that their alcohol consumption exceeded the recommended maximum drinking limit and were given information on the associated health and social risks. (2) The advice also included links for further standardised self-help material and a local alcohol treatment facility. | Received no intervention but were assessed at 6 and 12 months post-intervention. | 5 |
| Hester 1997 [17] | USA, recruited via Health Centres, newspaper advertisements, a screening program for drink-driving, radio, electronic bulletin boards, flyers around community and university (n=42; 36.3; 60%) | 3 (<10%) | Received Behavioural Self-Control Training, teaching skills in: (i) goal setting, (ii) self-monitoring, (iii) rate control and drink refusal, (iv) behavioural contracting with rewards and penalties, (v) evaluating triggers to over-drinking and problem solving to deal with them, (vi) functional analysis of drinking, and (vi) relapse prevention. The programme was delivered via computer over 10 weeks during 8 weekly therapist sessions ranging from 15 to 45 minutes each. 2 participants opted to take the diskette home with them for self-monitoring and upload the data during the therapist sessions. This intervention followed Miller and Mufioz's (1982) protocol for self-control training. | Received the same intervention after 10 weeks of waiting. | 16 |
| Hester 2005 [18] | USA, recruited via media advertisements, aged 21+ (n=61; 52%) | 1 (<10%) | Received a web-based intervention (the Drinker's Check-Up) via clinic-based computers for 90 minutes on average, based on AUDIT scores of 'at-risk' or higher (8+). (1) Participants were required to register their details so as to receive personalised responses from the program; it scored their risk, alcohol consumption and alcohol-related consequences as low, medium, high or very high. (2) The program integrated: (i) an assessment module with a decisional balance exercise comparing good/bad in drinking; (ii) a feedback module, in which gender, height and weight were used to calculate peak blood alcohol concentration (BAC) and assessments were compared to norms; and, (iii) a decision-making module, in which the participants' readiness to change was measured and the appropriate output provided. (3) Those that were assessed to be ready to change received assistance with planning and goal setting; those that were unsure received a second decisional balance exercise and those that were not ready only received the feedback report. | Received the same web-based intervention 4 weeks after the intervention group and were not assessed until then. | 19 |
| Hester 2012 (exp 1) [19] | USA, students recruited via college newspaper advertisements and flyers posted around campus, aged 18-24 (n=144) | 12 (around 10%) | Received the web-based CDCU (College Drinkers' Check-Up) intervention via computer for 35 minutes. The program provided an overview and also consisted of: (1) screening for heavy drinking using the AUDIT scale as well as 2 questions regarding the individual’s heaviest drinking in the last two weeks; (2) personalised feedback - those who screened positive for heavy drinking were invited to use the rest of the program following registration; (3) the Look at Your Drinking module which includes: (i) a decisional balance exercise, (ii) a comprehensive assessment of drinking and drug use, (iii) alcohol-related problems, and (iv) risk factors for future alcohol-related problems; (4) the Get Feedback module, which applies gender- and university-specific norms to provide feedback on (i) the quantity and frequency of their drinking compared to their same gender fellow students at their university, (ii) BAC feedback, and (iii) feedback on how their frequency of alcohol-related problems compares to other, same gender students at their school. (5) the Consider Your Options module which extends the initial decisional balance exercise, asking users to rate the level of importance of the “good things” and the “not so good things” about their drinking. Through this module, users could also receive help in developing a plan of action to reduce their drinking and risk for alcohol-related problems, provided they were ready to change their drinking. The CDCU was based on the original, face-to-face protocol by the same name that was developed by Miller and colleagues. | Received only the assessment module of the Web-based CDCU program via computer. | 18 |
| Hester 2012 (exp 2) [19] | USA, students, aged 18-24 (n=82) | 1 (around 10%) | Received the Web-based CDCU intervention via computer for 35 minutes. The program provided an overview and also consisted of: (1) screening for heavy drinking using the AUDIT scale as well as 2 questions regarding the individual’s heaviest drinking in the last two weeks; (2) personalized feedback - those who screened positive for heavy drinking were invited to use the rest of the program following registration; (3) the Look at Your Drinking module which includes: (i) a decisional balance exercise, (ii) a comprehensive assessment of drinking and drug use, (iii) alcohol-related problems, and (iv) risk factors for future alcohol-related problems; (4) the Get Feedback module, which applies gender- and university-specific norms to provide feedback on (i) the quantity and frequency of their drinking compared to their same gender fellow students at their university, (ii) BAC feedback, and (iii) feedback on how their frequency of alcohol-related problems compares to other, same gender students at their school. (5) the Consider Your Options module which extends the initial decisional balance exercise, asking users to rate the level of importance of the “good things” and the “not so good things” about their drinking. Through this module, users could also receive help in developing a plan of action to reduce their drinking and risk for alcohol-related problems, provided they were ready to change their drinking. The CDCU was based on the original, face-to-face protocol by the same name that was developed by Miller and colleagues. | Participants were not assessed until the 1-month follow-up when data was collected on: (i) their baseline drinking for the month prior to enrolling in the study (and alcohol-related problems in the previous year) and (ii) their drinking in the month between enrollment and follow-up. | 18 |
| Khadjesari 2014 [20] | UK, employees aged 18+ recruited via company web portal (n=1330; 75%) | 3 (around 20%) | Received an Internet-based lifestyle feedback intervention involving: (1) (i) screening in the form of an online health check that required details of their height and weight (for calculating Body Mass Index - BMI), alcohol consumption, smoking status, fruit and vegetable consumption and level of physical activity. (ii) feedback for BMI, which was grouped as underweight, healthy weight, overweight, obese or morbidly obese; this was accompanied by links to relevant NHS Choices webpages and the organisation’s own behaviour specific webpages. (iii) feedback on all health behaviours assessed in the health check including alcohol feedback, which provided criterion or risk-based feedback on the potential harm of drinking above recommended limits. (2) Optionally, an additional Web-based resource, Down Your Drink (DYD), was provided for participants who wanted help to reduce their drinking. DYD is an extended online alcohol intervention based on the principles of motivational interviewing, cognitive behavioural therapy, behavioural self-control, and relapse prevention (www.downyourdrink.org.uk). (3) Participants received feedback on their alcohol intake after completing the 3-month follow-up measures. | Received feedback on all health behaviours except alcohol consumption in a wait-list design. Participants received feedback on their alcohol intake after completing the 3-month follow-up measures. | 4 |
| Kypri 2009 [21] | Australia, students aged 17-24 (n=2435; 19.7; 55%) | 6 (35%; all participants included in the analysis through imputation and sensitivity analysis carried out) | Received an eSBI web-based intervention consisting of: (1) (i) an AUDIT score with an explanation of the associated health risk and information about how to reduce that risk; (ii) an estimated blood alcohol concentration (BAC) for the respondent’s heaviest episode in the previous 4 weeks, with information on the behavioural and physiological sequelae of various blood alcohol concentrations and traffic crash relative risk; (iii) estimates of monetary expenditure per month and year; (iv) bar graphs comparing episodic and weekly consumption with that of other students of the same age and sex; and (v) hyperlinks for smoking cessation and help with drinking problems. Three more optional web pages offered facts about alcohol and tips for reducing the risk of alcohol-related harm as well as provided information about where to find medical help and counselling support. (2) Following the 1-month assessment, participants received additional feedback comparing drinking levels that they reported at 1 month with those at baseline (a form of booster intervention). | Received no intervention but were screened. | 9 |
| Kypri 2013 [22] | New Zealand, Mauri students recruited via email aged 17-24 (n=1789; 20.2) | 5 (around 20%) | Received eSBI web-based assessment and personalised feedback on drinking via computer. (1) Participants' drinking habits were assessed using the AUDIT scale and the Leeds Dependency Questionnaire (LDQ). (2) Participants then received personalised feedback consisting of: (i) AUDIT score; (ii) LDQ score; (iii) explanation of associated health risk; (iv) information on how to reduce risk; (v) estimated BAC for respondents' heaviest drinking episode in the past 4-weeks; (vi) information on behavioural and psychological sequelae of various BACs; (vii) traffic crash relative risks; (viii) estimates of monetary expenditure in past month; (ix) bar graphs comparing episodic and weekly consumption with that of other students and members of general public (of same age and gender); (x) hyperlinks for help with drinking problems; and, (xi) web pages with general info/facts/medical help. | Received no intervention but were screened using the AUDIT-C tool; they subsequently filled in a brief questionnaire at the final 5-month follow-up. | 9 |
| Kypri 2014 [23] | New Zealand, students recruited via email, aged 17-24 (n=3422; 20.3) | 5 (around 20%) | Received eSBI web-based assessment and personalised feedback on drinking via computer. (1) Participants' drinking habits were assessed using the AUDIT scale and the Leeds Dependency Questionnaire (LDQ). (2) Participants then received personalised feedback consisting of: (i) AUDIT score; (ii) LDQ score; (iii) explanation of associated health risk; (iv) information on how to reduce risk; (v) estimated BAC for respondents' heaviest drinking episode in the past 4-weeks; (vi) information on behavioural and psychological sequelae of various BACs; (vii) traffic crash relative risks; (viii) estimates of monetary expenditure in past month; (ix) bar graphs comparing episodic and weekly consumption with that of other students and members of general public (of same age and gender); (x) hyperlinks for help with drinking problems; and, (xi) web pages with general information, facts and medical help. | Received no intervention but were screened using the AUDIT-C tool. | 8 |
| Labrie 2013 [24] | USA, students recruited via email, aged 18-24 (n=1831; 19.9; 43%) | 12 (around 10%; but very vague about why some participants with missing data were excluded and others were included in the analysis) | Received web-based feedback via email immediately after completing the 20 minute baseline survey. (1) Web-BASICS contained a total of 26 pages of interactive comprehensive motivational information addressing: (i) quantity and frequency of alcohol use; (ii) past-month peak alcohol consumption; (iii) estimated blood alcohol content (BAC), (iv) standard drink size, (v) how alcohol affects men and women differently, (vi) oxidation, (vii) alcohol effects, (viii) reported alcohol-related experiences, (ix) estimated calories and financial costs based on reported weekly use, (x) estimated level of tolerance, (xi) risks based on family history, (xii) risks for alcohol problems, (xiii) tips for reducing risks while drinking and, (xiv) alternatives to drinking. (2) The feedback also included PNF using typical student drinking norms. (3) Participants were given the option to click links throughout the feedback to obtain additional information on (a) standard drink size, (b) sex differences and alcohol use, (c) oxidation, (d) biphasic tips, (e) hangovers, (f) alcohol costs, (g) tolerance, (h) protective factors, and, (i) a link to a blood alcohol concentration (BAC) calculator. Web-BASICS was modelled from the in-person BASICS intervention. | Received generic nonalcohol-related normative feedback via email immediately after completing the 20 minute baseline survey. Information was provided on the typical student’s frequency of text messaging, downloading music, and playing video games on their campus. | 9 |
| Lewis 2007a [25] | USA, students recruited from psychology classes (n=185; 20.1; 45%) | 1 (not reported by arm) | Received gender-specific PNF (personalised normative feedback) via computer for 1-2 minutes following baseline assessment. This feedback was then provided as a printout to take away but was not further discussed, except in situations when comments were made or questions asked about PNF. (1) Information was provided on: (i) personal drinking, (ii) perceptions of typical student drinking, and (iii) actual typical student drinking norms. Information pertaining to perceptions of typical student drinking and actual typical student drinking norms provided a discrepancy suggesting to heavy-drinking students that “most students don’t drink as much as you think they do.” Feedback relating to personal drinking behaviour and actual typical student drinking norms provided students with a discrepancy pointing out to heavy drinking students that “most students don’t drink as much as you do.” Actual typical student drinking behaviour norms were based on screening data. (2) Participants’ percentile ranking comparing their drinking with that of other students was also provided. This intervention was modelled on BASICS. | Did not receive any intervention and were only assessed. | 2 |
| Lewis 2007b [26] | USA, students recruited from a freshman orientation class via telephone or email (n=245; 18.53; 48%) | 5 (<20%) | Received gender-specific PNF personalised normative feedback targeted at freshmen via the Web, initially for 60 minutes (at baseline). (1) The feedback consisted of information on: (i) personal drinking behaviour, (ii) personal perceptions of typical student drinking behaviour, and (iii) actual norms for typical student drinking behaviour. Actual norms for typical student drinking behaviour creates two discrepancies for heavy-drinking students when compared with personal drinking behaviour (i.e., most students don't drink as much as you do) and personal perceptions of typical student drinking behaviour (i.e., most students don't drink as much as you think they do). Participants in this arm received gender-specific norms based on responses from a screening survey. (2) Students receiving PNF were also provided with the percentile rank of their drinking in comparison to other students. | Did not receive any intervention and were only assessed. | 2 |
| Lewis 2014 [27] | USA, undergraduate students recruited via email or letter, aged 18-25 (n=480; 20.08; 42%) | 6 (<20%) | All pages contained a banner with the study logo that read “How do you compare to other male/female [university name] students?” The PNF included information regarding (a) one’s own behaviour, (b) one’s perceptions of the typical same-sex students’ behaviour, and (c) the typical same-sex students’ actual behaviour (i.e., the campus norm). This information was presented in text and bar graph format. Each screen presented one graph and related feedback content. The final screen of the feedback provided a percentile rank for comparison between the participants’ reported drinking and that of their same-sex peers. This intervention was extremely brief. | Shown information related to use of technology (3 screens). Technology use was broken down into three topics: (a) texting, (b) downloading music, and (c) playing video games. Each screen presented one graph and related feedback content. For each screen of the feedback, participants were provided their percentile rank for the specific technology uses. Duration 1-5 minutes. | 2 |
| Murphy 2010 [28] | USA, students aged 18+ recruited via the university health clinic (n=133; 18.6; 50%) | 1 (around 10%) | Received e-CHUG, an interactive web-based program that requires students to complete a brief drinking assessment (6-7 minutes) that is used to instantly generate personalised feedback in the following areas: (a) quantity and frequency of drinking, (b) comparison of drinking with student norms, (c) peak BAC, (d) tolerance level, (e) alcohol related consequences, (f) money spent on alcohol, (g) calories consumed from alcohol, and (h) family risk score. Students were asked to review the feedback for at least 30 minutes and completed a brief comprehension check to ensure adequate exposure to the intervention. Duration at least 35 minutes. | Received computerised assessment only. | 11 |
| Neighbors 2006 [29] | USA, students recruited from a psychology class (n=214; 19.67; 44%) | 2 (<20%) | Received PNF delivered via computer. All participants were thanked for their participation and were informed that they would be contacted at a later date to schedule an appointment for follow-up assessment. Procedures for follow-up assessment were similar, with the exception that no feedback was provided. Upon completion of follow-up assessment, participants were provided with a written debriefing that explained the purpose and design of the study. | Received computerised assessment only. | 2 |
| Neumann 2006 [30] | Germany, recruited from an emergency department with subcritical injuries; aged 18+ (n=1139) | 12 (>30%) | Received computer-generated feedback about current drinking status based on information obtained from the AUDIT and RTC-Q. The results were displayed on the computer, and a letter summarising the intervention was then printed and provided to the patient before discharge from the ED. The written intervention contained feedback about the level of alcohol consumption compared with safe drinking norms, and emphasised personal responsibility for determining the need for change. It provided clear advice about the need to change drinking patterns and to develop goals for behavioural change. A menu of alternative strategies for changing alcohol consumption patterns, including treatment assisted change or self-change, was provided. The information was presented in a respectful, empathic manner that was meant to increase the level of motivation for change, and to increase the patient’s sense of self-efficacy and optimism. The elements of the intervention can be summarised with the acronym FRAMES: Feedback, Responsibility, Advice, Menu, Empathy, Self-efficacy. Feedback and information was provided concerning each positive AUDIT question. Patients’ level of motivation was also incorporated into the intervention. Additional educational information was provided regarding risky situations and drinking triggers that should be avoided, contraindications to alcohol use, and symptoms of dependence. A list of alcohol treatment services available in the community was also provided. To reduce resistance and stigmatisation, feedback concerning alcohol was embedded with information about other lifestyle risks, including diet, tobacco and drug use, and other risky behaviours. | Received care for their injuries only. | 12 |
| Postel 2010 [31] | Netherlands, recruited via advertisements on websites and national media, aged 18+ (n=156; 45.3; 46%) | 3 (large differential in loss to follow-up between groups) | Received a structured 2-part e-therapy online treatment program in which the participant and the therapist communicated asynchronously, via the Internet only. Participants accessed the e-therapy program in their personal environment. Participant and therapist were in separate or remote locations; the interaction occurred with a time delay between the responses. The aim of the e-therapy program was to reduce or stop the participant’s alcohol intake. All communication between therapists and participants took place through a Web-based application. Part 1 of the program consisted of 2 assessments and 4 assignments, with the accompanying communication focusing on the analysis of the participants’ drinking habits. Part 2 focused on behavioural change and included 5 central concepts: (1) setting a drinking goal, which could be abstinence or moderate drinking, (2) formulating helpful and non helpful thoughts, (3) considering helpful behaviours for moments of craving, (4) identifying the moment of the decision to drink alcohol, and (5) formulating an action plan for maintaining the new drinking behaviour and for preventing relapse. Duration: participants registered daily with the program and had 2-3 therapist contacts a week for 3 months; therapists spent 1.5 hrs per week. | On waiting list; received no-reply email messages containing alcohol-related information, psychoeducational material, motivational messages, and references to the information website and the forum for online contact with fellow sufferers. | 12 |
| Riper 2008 [32] | Netherlands, recruited via advertisements in newspaper and health websites, aged 18-65 (n=261; 46; 51%) | 6 (>40%) | Received the Drinking Less intervention which consists of a homepage giving information on alcohol and treatment services, and offering access to the self-help programme via an automated sign-up procedure, with a description indicating for whom the intervention is suitable. The self-help program proceeds in four successive stages: preparing for action, goal setting, behavioural change, and maintenance of gains and relapse prevention. The self-help program also contains a moderated peer-to-peer discussion forum. Trial participants were allowed to use the intervention for as long as they felt necessary (24 hours a day, 7 days per week access throughout the trial period). | Received access to a web-based psychoeducational brochure on the effects of alcohol use, which described the impact of alcohol use on physical and social functioning in a factual manner and took approximately 15 minutes to read. | 5 |
| Schulz 2013 [33] | Germany, recruited via an online access panel, aged 18-69 (n=448; 41.72; 57%) | 6 (>30%) | The web-based intervention *Alcohol - Everything Within the Limits?!* was in 5 parts: Part 1 served as a starting point of the drinking behaviour change process (premotivational phase) by addressing the concepts of knowledge and awareness: it gave information about the German alcohol guidelines and assessed whether respondents were meeting them by using comparative/normative feedback. In addition, respondents’ scores were depicted graphically using a traffic light symbol (indicating whether they met, almost met, or did not meet the guidelines). To increase the respondent’s level of knowledge, the relation between alcohol and various diseases was explained, and information tailored to the respondent’s health status was given about alcohol and pregnancy, and about the possible influence of participants’ drinking behaviour on their children (if applicable). Part 2 offered personalised feedback concerning the perceived pros and cons of alcohol drinking as perceived by the respondent, with the goal of creating a positive attitude toward not drinking more than 1 (women) or 2 (men) alcoholic drinks per day. Part 3 explained the importance of social influence in a tailored message by focusing on the respondent’s partner, family, friends, and colleagues. In the fourth part, preparatory action plans were defined to prepare the intended behavioural change. The final part focused on self-efficacy and coping plans by identifying difficult situations and suggesting ways to cope with them. Personalised tips were given on how to deal with the perceived difficult situations to overcome potential barriers (postmotivational phase), and the situations and plans were summarised for individual respondents to help them remember these. Duration not reported. | Web-based assessment only. | 9 |
| Sugarman 2009 [34] | USA, students recruited in psychology classes and through flyers, aged 18+ (n=393; 45%) | 2 (45%) | Received a single session of personalised feedback on (i) the quantity and (ii) the frequency of their drinking, (iii) the frequency of heavy drinking episodes, (iv) their average BAC and (v) their peak BAC, and (vi) a list of any alcohol related problems that they reported experiencing in the past month. In addition, this information was presented in the context of (vii) national and (viii) local (Syracuse University) normative gender-specific data. The feedback also contained (i) educational information on BAC, (ix) the effects of alcohol on the body, and (x) tips for safer drinking. Duration average 2 minutes. | Received general health information. | 7 |
| Voogt 2013a [35] | Netherlands, recruited via their vocational education school, aged 15-20 (n=609; 17.3; 60%) | 6 (>40%) | Received the WDYD (What Do You Drink?) intervention: a single session web-based brief alcohol intervention to detect and reduce heavy drinking of adolescents. The WDYD intervention, developed by using the IM protocol, is based on Motivational Interviewing principles and elements of the I-Change model. Knowledge, social norms and self-efficacy are embedded in the intervention as the most changeable determinants of behaviour change. Duration 20 minutes. | Assessed only. | 10 |
| Voogt 2013b [36] | Netherlands, students recruited via flyers distributed around campus, aged 18-24 (n=913; 20.8; 60%) | 6 (around 10%) | The first part of WDYD (What Do You Drink?) focuses on the motivation phase of the behaviour change process and contains a homepage and a screening test with personalised feedback. The screening test includes items addressing participants’ name, sex, age, education level, weight, alcohol use, willingness to change alcohol consumption, average expenses on consumed alcohol beverages, and descriptive social norms. After completing the screening test, participants will receive personalised feedback tailored to participants’ sex, alcohol intake, and perceived social norm. It will provide 1) advice about drinking according to the guidelines of the Dutch National Health Council. It will provide information about 2) the amount of glasses of standard alcohol units that the participant consumed in the last year, with estimates of the number of calories consumed, the amount of weight added because of drinking, and the amount of money spent on drinking. Lastly, it will depict 3) a bar chart comparing the number of glasses of standard alcohol units per week that participants think their same-sex peers consume with the number of glasses of standard alcohol units per week that participants’ same-sex peers actually consume. The second part of WDYD focuses on the action phase of the behaviour change process, with a general goal of reducing heavy drinking. Participants will be prompted to make decisions about the maximum amount of glasses of standard alcohol units they want to drink on every day of the week at a given point of time, preferably within the limits of low-risk drinking. WDYD focuses on strengthening participants’ drinking refusal self-efficacy by proving tips to resist alcohol in different drinking situations. Duration 20 minutes. | No intervention. | 11 |
| Wagener 2012 [37] | USA, student members of an online university participant pool management system, aged 18-26 (n=152; 20.9; 55%) | 3 (<10%) | Received DrAFT-CS (Drinking Assessment and Feedback Tool for College Students), included a CA of alcohol use behaviours, consequences, and perceived norms followed immediately by on-screen personalised feedback. The assessment included measures of quantity and frequency of drinking, common problems experienced by college drinkers, levels of alcohol dependence, perceptions of drinking norms, perceptions of alcohol-related risk, overall levels of psychological distress, and motivation for change in drinking behaviours. The personalised feedback included quantity and frequency of use; typical and peak blood alcohol levels achieved on drinking occasions; perceptions of social norms; dependence criteria; alcohol-related problems experienced; financial and caloric costs of alcohol use; familial risk for alcohol problems; perceptions of risk; alcohol expectancies; psychological problems, such as depression and anxiety, that may exacerbate or contribute to alcohol abuse; and motivation for changing current alcohol use. To simulate face-to-face PFIs and enhance interest and engagement in the program, the DrAFT-CS also includes a video interviewer. The interviewer appears periodically as the user progresses through the program and offers a welcome message, provides instructions for assessments, offers encouragement, and provides interpretive information for feedback screens. This interviewer is a unique component of the DrAFT-CS that is not found in any other computer delivered PFIs that are primarily text based. The interviewer was also designed to provide information in an empathic, nonjudgmental manner consistent with principles of MI. Duration 45 minutes. | Completed computer-based assessment only. | 12 |
| Wallace 2011 [38] | UK, recruited via advertisements on the Alcohol Concern website or through finding the study via search engines, aged 18+ (n=2652) | 12 (>60%) | Had access to DYD (Down Your Drink), a theoretically informed web-based programme, based on brief intervention and psychological treatment principles. It offered three phases, each of which was divided into levels with different materials and associated exercises and tasks. If followed in order they provided a natural progression through three stages: decision making (Phase 1, ‘‘It’s up to you’’); implementing change (Phase 2, ‘‘Making the change’’); and relapse prevention (Phase 3, ‘‘Keeping on track’’). However, users were free to design their own route through the programme, and could use it as often or as seldom as they wished. Phase 1 was based on the principles of motivational enhancement therapy, phase 2 used computerised cognitive behavioural therapy and behavioural self control principles, and phase 3 was based on principles of relapse prevention. There were a number of interactive ‘‘e-tools’’ including a ‘‘thinking drinking diary’’ in which users could record their alcohol consumption along with emotional and behavioural triggers and responses. Duration not reported. | Had access to a comparator website used a similar graphical design and style as the intervention website to present simple, text-based information about the harms caused by excess alcohol consumption. It did not contain any interactive components, and users did not have access to the e-tools. For the duration of the trial, this comparator website was also referred to as DownYourDrink so that participants were not aware whether they had access to the intervention or comparator site. | 22 |
| Walters 2009 [39] | USA, students recruited via class presentations, email and flyers distributed across campus, aged 18+ (n=279; 19.8; 36%) | 6 (<20%) | Had access to e-CHUG. Using the information from a participant’s assessment, the feedback included: (1) a quantity/frequency summary of drinking behaviour (e.g., standard drinks consumed in the last 30-days, estimated peak BAC, caloric intake), (2) comparison to U.S. adult and campus norms, (3) level of risk (e.g., AUDIT score, tolerance, estimated genetic risk), (4) estimated dollar amount and percent of income spent on alcohol, and (5) local referral resources. Duration not reported. | Completed web-based assessment only. | 13 |
| Weaver 2014 [40] | USA, students recruited from an undergraduate psychology class, aged 18-25 (n=176; 51%) | 1 (around 20%) | Had access to DrAFT-CS, beginning with a video clinician who explained the intent of the program. Participants were guided by the video clinician through questions that assessed a variety of variables necessary for personalised feedback. Participants answered questions that assessed quantity and frequency of alcohol use, alcohol-related problems, alcohol dependence symptoms, perceptions of drinking norms, psychological distress, and motivation to change drinking behaviour. Once participants completed the assessment phase of the DrAFT-CS, the video clinician introduced the feedback section of the intervention and provided personalised feedback regarding their alcohol use, alcohol-related problems, risk for developing an alcohol use disorder, normative feedback on drinking behaviour, money spent on alcohol, and calories consumed. Unique to DrAFT-CS, participants received this information via the video clinician who explained each piece of feedback as it was presented. Consistent with the style of motivational interviewing (Miller & Rollnick, 2002), the video clinician presented information without suggesting a need for change in order to reduce the risk of resistance from the participant. Once the feedback was completed, the research assistant closed the program and informed the participant that they would be contacted via email with a link to complete follow-up assessments. The DrAFT-CS took approximately 45-minutes to complete. | Performed computer-based assessment only. | 8 |

Full references for included studies:

1. Bendtsen P, Bendtsen M, Karlsson N, et al. Online alcohol assessment and feedback for hazardous and harmful drinkers: Findings from the AMADEUS-2 randomized controlled trial of routine practice in Swedish universities. *J Med Internet Res* 2015;**17**:e170.

2. Bertholet N, Cunningham JA, Faouzi M, et al. Internet-based brief intervention for young men with unhealthy alcohol use: A randomized controlled trial in a general population sample. *Addiction* 2015;**110**(11):1735-1743.

3. Blankers M, Koeter MW, Schippers GM. Internet therapy versus internet self-help versus no treatment for problematic alcohol use: A randomized controlled trial. *J Consult Clin Psychol* 2011;**79**:330-41.

4. Brendryen H, Lund IO, Johansen AB, et al. Balance-a pragmatic randomized controlled trial of an online intensive self-help alcohol intervention. *Addiction* 2014;**109**:218-26.

5. Brief DJ, Rubin A, Keane TM, et al. Web intervention for OEF/OIF veterans with problem drinking and PTSD symptoms: A randomized clinical trial. *J Consult Clin Psychol* 2013;**81**:890-900.

6. Butler LH, Correia CJ. Brief alcohol intervention with college student drinkers: Face-to-face versus computerized feedback. *Psychology of Addictive Behaviors* 2009;**23**:163-7.

7. Chiauzzi E, Green TC, Lord S, et al. My student body: A high-risk drinking prevention web site for college students. *Journal of American College Health* 2005;**53**:263-74.

8. Collins SE, Kirouac M, Lewis MA, et al. Randomized controlled trial of web-based decisional balance feedback and personalized normative feedback for college drinkers. *J Stud Alcohol Drugs* 2014;**75**:982-92.

9. Cunningham JA, Wild TC, Cordingley J, et al. Twelve-month follow-up results from a randomized controlled trial of a brief personalized feedback intervention for problem drinkers. *Alcohol Alcohol* 2010;**45**:258-62.

10. Delrahim-Howlett K, Chambers CD, Clapp JD, et al. Web-based assessment and brief intervention for alcohol use in women of childbearing potential: A report of the primary findings. *Alcohol Clin Exp Res* 2011;**35**:1331-8.

11. Doumas DM, Haustveit T, Coll KM. Reducing heavy drinking among first year intercollegiate athletes: A randomized controlled trial of web-based normative feedback. *Journal of Applied Sport Psychology* 2010;**22**:247-61.

12. Doumas DM, Kane CM, Navarro B, et al. Decreasing heavy drinking in first-year students: Evaluation of a web-based personalized feedback program administered during orientation. *Journal of College Counseling* 2011;**14**:5-20.

13. Ekman DS, Andersson A, Nilsen P, et al. Electronic screening and brief intervention for risky drinking in Swedish university students - a randomized controlled trial. *Addict Behav* 2011;**36**:654-9.

14. Gajecki M, Berman AH, Sinadinovic K, et al. Mobile phone brief intervention applications for risky alcohol use among university students: A randomized controlled study. *Addict Sci Clin Pract* 2014;**9**:11.

15. Geisner IM, Varvil-Weld L, Mittmann AJ, et al. Brief web-based intervention for college students with comorbid risky alcohol use and depressed mood: Does it work and for whom? *Addict Behav* 2015;**42**:36-43.

16. Hansen AB, Becker U, Nielsen AS, et al. Internet-based brief personalized feedback intervention in a non-treatment-seeking population of adult heavy drinkers: A randomized controlled trial. *J Med Internet Res* 2012;**14**:e98.

17. Hester RK, Delaney HD. Behavioral self-control program for windows: Results of a controlled clinical trial. *J Consult Clin Psychol* 1997;**65**:686-93.

18. Hester RK, Squires DD, Delaney HD. The drinker's check-up: 12-month outcomes of a controlled clinical trial of a stand-alone software program for problem drinkers. *Journal of Substance Abuse Treatment* 2005;**28**:159-69.

19. Hester RK, Delaney HD, Campbell W. The college drinker's check-up: Outcomes of two randomized clinical trials of a computer-delivered intervention. *Psychology of Addictive Behaviors* 2012;**26**:1-12.

20. Khadjesari Z, Freemantle N, Linke S, et al. Health on the web: Randomised controlled trial of online screening and brief alcohol intervention delivered in a workplace setting. *PLoS One* 2014;**9**:e112553.

21. Kypri K, Hallett J, Howat P, et al. Randomized controlled trial of proactive web-based alcohol screening and brief intervention for university students. *Arch Intern Med* 2009;**169**:1508-14.

22. Kypri K, McCambridge J, Vater T, et al. Web-based alcohol intervention for Maori university students: Double-blind, multi-site randomized controlled trial. *Addiction* 2013;**108**:331-8.

23. Kypri K, Vater T, Bowe SJ, et al. Web-based alcohol screening and brief intervention for university students: A randomized trial. *Jama* 2014;**311**:1218-24.

24. Labrie JW, Lewis MA, Atkins DC, et al. RCT of web-based personalized normative feedback for college drinking prevention: Are typical student norms good enough? *J Consult Clin Psychol* 2013;**81**:1074-86.

25. Lewis MA, Neighbors C. Optimizing personalized normative feedback: The use of gender-specific referents. *J Stud Alcohol* 2007;**68**:228-37.

26. Lewis MA, Neighbors C, Oster-Aaland L, et al. Indicated prevention for incoming freshmen: Personalized normative feedback and high-risk drinking. *Addict Behav* 2007;**32**:2495-508.

27. Lewis MA, Patrick ME, Litt DM, et al. Randomized controlled trial of a web-delivered personalized normative feedback intervention to reduce alcohol-related risky sexual behavior among college students. *Journal of Consulting and Clinical Psychology* 2014;**82**:429–40.

28. Murphy JG, Dennhardt AA, Skidmore JR, et al. Computerized versus motivational interviewing alcohol interventions: Impact on discrepancy, motivation, and drinking. *Psychology of Addictive Behaviors* 2010;**24**:628-39.

29. Neighbors C, Lewis MA, Bergstrom RL, et al. Being controlled by normative influences: Self-determination as a moderator of a normative feedback alcohol intervention. *Health Psychol* 2006;**25**:571-9.

30. Neumann T, Neuner B, Weiss-Gerlach E, et al. The effect of computerized tailored brief advice on at-risk drinking in subcritically injured trauma patients. *J Trauma* 2006;**61**:805-14.

31. Postel MG, de Haan HA, ter Huurne ED, et al. Effectiveness of a web-based intervention for problem drinkers and reasons for dropout: Randomized controlled trial. *J Med Internet Res* 2010;**12**:e68.

32. Riper H, Kramer J, Smit F, et al. Web-based self-help for problem drinkers: A pragmatic randomized trial. *Addiction* 2008;**103**:218-27.

33. Schulz DN, Candel MJ, Kremers SP, et al. Effects of a web-based tailored intervention to reduce alcohol consumption in adults: Randomized controlled trial. *J Med Internet Res* 2013;**15**:e206.

34. Sugarman, DE. Web-based alcohol feedback intervention for heavy drinking college students: does drinking control strategy use mediate intervention effects?. PhD Thesis, Syracuse University 2009.

35. Voogt CV, Kleinjan M, Poelen EAP, et al. The effectiveness of a web-based brief alcohol intervention in reducing heavy drinking among adolescents aged 15-20 years with a low educational background: A two-arm parallel group cluster randomized controlled trial. *BMC Public Health* 2013;**13**:11.

36. Voogt CV, Poelen EA, Kleinjan M, et al. The effectiveness of the 'What Do You Drink' web-based brief alcohol intervention in reducing heavy drinking among students: A two-arm parallel group randomized controlled trial. *Alcohol Alcohol* 2013;**48**:312-21.

37. Wagener TL, Leffingwell TR, Mignogna J, et al. Randomized trial comparing computer-delivered and face-to-face personalized feedback interventions for high-risk drinking among college students. *Journal of Substance Abuse Treatment* 2012;**43**:260-7.

38. Wallace P, Murray E, McCambridge J, et al. On-line randomized controlled trial of an internet based psychologically enhanced intervention for people with hazardous alcohol consumption. *PLoS ONE* 2011;**6**:e14740.

39. Walters ST, Vader AM, Harris TR, et al. Dismantling motivational interviewing and feedback for college drinkers: A randomized clinical trial. *J Consult Clin Psychol* 2009;**77**:64-73.

40. Weaver CC, Leffingwell TR, Lombardi NJ, et al. A computer-based feedback only intervention with and without a moderation skills component. *Journal of Substance Abuse Treatment* 2014;**46**:22-8.
